# The pharmacodynamic modulation effect of oxytocin on resting state functional connectivity network topology

**DOI:** 10.3389/fphar.2024.1460513

**Published:** 2025-01-06

**Authors:** Abraham Tonny Hagan, Lei Xu, Benjamin Klugah-Brown, Jialin Li, Xi Jiang, Keith M. Kendrick

**Affiliations:** MOE Key Laboratory for Neuroinformation, The Clinical Hospital of Chengdu Brain Science Institute, University of Electronic Science and Technology of China, Chengdu, China

**Keywords:** oxytocin, resting state fMRI, small-worldness, graph theory, pharmacodynamics

## Abstract

**Introduction:**

Neuroimaging studies have demonstrated that intranasal oxytocin has extensive effects on the resting state functional connectivity of social and emotional processing networks and may have therapeutic potential. However, the extent to which intranasal oxytocin modulates functional connectivity network topology remains less explored, with inconsistent findings in the existing literature. To address this gap, we conducted an exploratory data-driven study.

**Methods:**

We recruited 142 healthy males and administered 24 IU of intranasal oxytocin or placebo in a randomized controlled double-blind design. Resting-state functional MRI data were acquired for each subject. Network-based statistical analysis and graph theoretical approaches were employed to evaluate oxytocin’s effects on whole-brain functional connectivity and graph topological measures.

**Results:**

Our results revealed that oxytocin altered connectivity patterns within brain networks involved in sensory and motor processing, attention, memory, emotion and reward functions as well as social cognition, including the default mode, limbic, frontoparietal, cerebellar, and visual networks. Furthermore, oxytocin increased local efficiency, clustering coefficients, and small-world propensity in specific brain regions including the cerebellum, left thalamus, posterior cingulate cortex, right orbitofrontal cortex, right superior frontal gyrus, left inferior frontal gyrus, and right middle orbitofrontal cortex, while decreasing nodal path topological measures in the left and right caudate.

**Discussion:**

These findings suggest that intranasal oxytocin may produce its functional effects through influencing the integration and segregation of information flow within small-world brain networks, particularly in regions closely associated with social cognition and motivation.

## Introduction

The human brain has an intricate network of interconnected regions which form the functional connectome that is fundamental to how it processes all aspects of information influencing complex sensory, motor, cognitive and emotional behaviors (Sporns, 2011a; Van den Heuvel et al., 2012; Cromwell et al., 2020; Wasserman and Wasserman, 2023). Dysfunctions in these neural connections have been associated with neurological, mental and psychiatric disorders, including anxiety, autism, depression, schizophrenia, epilepsy and Alzheimer’s disease (Marín, 2012; Jiang et al., 2023) and it is important to identify potential therapeutic interventions.

Oxytocin (OT), a hypothalamic neuropeptide hormone, has garnered significant interest as a neuromodulator in cognitive processes due to its involvement in social cognition and motivation (Kendrick et al., 2018; Quintana et al., 2021) and recent clinical trials reporting improved social functioning in individuals with autism spectrum disorder (Audunsdottir et al., 2024; Le et al., 2022). OT receptors are predominantly found in key regions of the social brain, including the hippocampus, cingulate cortex, insula, amygdala, basal ganglia and medial prefrontal cortex (Gimpl and Fahrenholz, 2001; Quintana et al., 2019) and there is considerable interest in establishing the effects of OXT administration on these regions and their functional connections. Resting-state fMRI (rsfMRI) is a valuable technique for investigating intrinsic brain connectivity independently of specific tasks (Canario et al., 2021). By analyzing spontaneous fluctuations in blood oxygenation level dependent (BOLD) signals, rsfMRI allows us to map functional interactions between brain regions and explore how these interactions are influenced by interventions or pharmacological manipulations (Snyder, 2022; Smitha et al., 2017).

A number of studies have examined the effects of intranasal administration of OT on resting state functional connectivity, although findings have been variable (Seeley et al., 2018). One fairly consistent finding has been for increased functional connectivity between the medial frontal cortex and the amygdala (Eckstein et al., 2017; Kou et al., 2022; Kovács and Kéri, 2015; Sripada et al., 2013). This pathway is important for emotional control and several clinical studies have also reported that OT can either increase resting state functional connectivity between frontal regions and the amygdala in autism (Procyshyn et al., 2022) and social anxiety (Dodhia et al., 2014) or in post-traumatic stress disorder (Frijling et al., 2016; Koch et al., 2016). Other studies have reported effects of intranasal oxytocin on amygdalo-hippocampal functional connectivity in either healthy (Coenjaerts et al., 2023) or autistic (Alaerts et al., 2019) individuals. However, these studies were mostly region of interest based and studies at the whole brain level have reported more extensive resting state changes in involving frontal and striatal reward processing regions and the cerebellum (Zhao et al., 2019a), with weakened functional connectivity between frontal and striatal and motor regions also being found to be improved in schizophrenia patients (Korann et al., 2022). Additionally, a large-scale effective connectivity study using resting state data has found increased connectivity following intranasal OT between frontal, salience, social cognition, reward and amygdala networks (Jiang et al., 2021), although a very small scale one only found effects on the effective connectivity between the dorsolateral prefrontal cortex and the precuneus in the central executive network (Kumar et al., 2020). The effects of OT on resting state networks may also be influenced by OT receptor genotype (Kou et al., 2022; Seeley et al., 2018) Overall, therefore findings investigating resting state-based changes based on conventional analysis of time-series in region pairs has produced varied results.

Several studies have attempted to establish more network based effects of intranasal OT using independent component analysis of resting state data, although again with somewhat different findings. Two studies have reported that OT particularly increased functional connectivity between attention and salience and default mode networks, implying modulation of attentional processing (Brodmann et al., 2017; Xin et al., 2021) while another found evidence for increased functional connectivity involving the mentalizing network (temperoparietal junction) and the default mode network, but decreased between it and the medial prefrontal network (Wu H. et al., 2020).

Graph-theoretical techniques may offer an alternative robust framework for quantifying the effects of OT on both global and local properties of brain networks (Wig et al., 2011; Lang et al., 2012; Fornito et al., 2016). By modeling the brain as a graph, with nodes representing brain regions and edges representing functional connections, this technique can examine how OT influences efficiency, resilience, and integration within the brain using measures, including small-world propensity, clustering coefficient, characteristic path length, global efficiency, and local efficiency (Bassett and Bullmore, 2017). However, one particular aspect of brain connectivity that has gained significant attention in recent years is the concept of “small-world networks.” The human brain exhibits as a small-world network topology, characterized by a delicate balance between local clustering and short path length. This organization enables effective information processing while minimizing wiring costs (Sporns, 2011b). Disruptions in small-world network topology are associated with various neuropsychiatric disorders, including autism spectrum disorder, schizophrenia, and depression (Cao et al., 2015). Notably, modularity and clustering coefficients are also frequently used to quantify topological segregation properties in brain networks (Boccaletti et al., 2006; Rubinov and Sporns, 2010). The clustering coefficient, a frequently employed metric in brain research, serves as a representation of brain topology (Bullmore and Sporns, 2009) and several studies have established a correlation between mental illness, cognitive function, and this coefficient (Sæther et al., 2023; Ohi et al., 2017). Additionally, the average shortest path within neural networks, a prevalent indicator of the brain’s small-world characteristics, mirrors its capacity for efficient information integration.

Several small scale graph theoretical studies have suggested that OT may modulate regional but not global processing to influence integration of networks associated with cognitive, attention, theory of mind and sensory processing (Martins et al., 2021; Zheng et al., 2021; Davies et al., 2024). However, findings from these small studies have been variable and it is important to implement this powerful approach in a larger cohort of subjects in order to establish more robustly what influence OT has on modulating both regional and global network processing and the precise nature of this in terms of integration within different systems.

In the current study we have therefore carried out a whole-brain resting state study on a large cohort of healthy subjects (n = 142) employing a graph-theoretical methodology to establish the pharmacodynamic effects of intranasal OT on brain functional connectivity network topology. Our hypothesis based on previous findings was that intranasal OT would influence the integration and segregation of information within the graph measures and functional connectivity patterns of the brain.

## Materials and methodology

### Participants

139 non-smoking healthy adult male subjects were recruited via advertisement at the University of Electronic Science and Technology of China. Only males were recruited for the current study in line with most other OT resting state studies and to avoid potential complications in controlling for menstrual cycle effects in females. In terms of inclusion and exclusion criteria, all subjects were right-handed and self-reported having no current or previous mental health problems, neurological disorders or other medical conditions. They were instructed not to consume alcohol or caffeine in the last 24 h prior to the start of the study. To control for group differences in psychological traits, anxiety and mood, all participants completed the Becks Depression Inventory (BDI-2) (Beck et al., 1996), State-Trait Anxiety Inventory (STAI) (Spielberger, 1983), Positive and Negative Affect Schedule, (PANAS) (Watson et al., 1988), autism spectrum quotient (ASQ) (Baron-Cohen et al., 2001) and Empathy quotient (EQ) (Allison et al., 2011). All subjects gave written informed consent and the study was approved by the University of Electronic Science and Technology of China ethical committee. The study protocol was in accordance with the latest revision of the declaration of Helsinki.

### Study design

The study employed a randomized OT–placebo (PLC) controlled double blind between group design where each participant was randomly assigned to administer either OT or PLC intranasally.

### Procedure

Test sessions were conducted by trained study staff. Participants were instructed to stay well hydrated before their visits but to abstain from caffeine, alcohol, and use of recreational drugs in the 24 h, leading up to their appointment. Participants were randomly assigned using a computer-based algorithm to self-administer either 24 IUs (three puffs per nostril given 30 s apart) of OT (OT plus glycerine, sodium chloride and sterile water; Sichuan Defeng Pharmaceutical Co. Ltd., Sichuan, China) or PLC (identical ingredients as the OT spray except for OT, also provided by Sichuan Defeng Pharmaceutical Co. Ltd., Sichuan, China) at the start of the full study visit as this dosage is well established, maintaining safety, efficacy, comparability and consistency across studies while ensuring reproducibility of our findings in the broader oxytocin research community which aligns with a number of previous OT-administration studies (Frijling et al., 2016; Koch et al., 2016; Parker et al., 2017; Yao et al., 2017) nasal spray administration followed standard recommendations (Guastella et al., 2013) and the rsfMRI scan started 45 min after the nasal sprays. Participants completed the questionnaires immediately prior to administering the nasal spray. In the scanner, participants were instructed to relax with open eyes but without falling asleep and without thinking of anything in particular (Patriat et al., 2013; Raimondo et al., 2021).

### fMRI acquisition

MRI data were collected on a 3T GE MR750 Discovery MRI system (General Electric Medical System, Milwaukee, WI, USA). Functional gradient-echo-planar imaging (EPI) data were acquired during the resting-state paradigm (38 interleaved slices, TR 2s, TE 30 ms, FOV 252 × 252 × 133 mm, 80 × 80 × 38 mm matrix, flip angle 90°, in plane resolution of 3.15 × 3.15 mm, slice thickness 3.5 mm, 0 mm skip). The resting state scan lasted about 8 min, and 240 time points were acquired. Whole-brain high-resolution three-dimensional T1-weighted anatomical reference images were also acquired using an MP-RAGE sequence (sagittal plane, FOV = 240 mm × 240 -mm × 170; 1 × 1 × 1 mm isotropic voxels). Processing and analysis of brain images were performed using Statistical Parametric Mapping (SPM8) software (www.fifil.ion.ucl.ac.uk/spm).

### fMRI data preprocessing

Standard preprocessing procedures were employed including slice time correction, motion correction with artifact rejection (scan-to-scan motion threshold 2 mm), spatial normalization, and smoothing with an 8 mm Gaussian kernel as implemented in the Functional Connectivity Toolbox (CONN; http://www.nitrc.org/projects/conn/; Whitfield-Gabrieli and Nieto-Castanon, 2012). Prior to analysis, data were denoised using “aCompCor,” an anatomically informed component-based noise correction, to correct for physiological and other sources of noise from white matter and cerebrospinal fluid (Behzadi et al., 2007).

### Data analysis

The study comprised two treatment groups (OT (N = 67, aged 22.09 ± 2.44 years) and PLC (N = 72 aged 21.69 ± 2.57 years) in a between-group design. The data from the 139 participants were analyzed and the brain signals extracted from the 116 brain regions according to the Automated Anatomical Labelling (AAL) brain atlas. To determine the effects of OT on the intrinsic connectivity among brain regions, Pearson correlation of mean fMRI time series between any pair of the 116 regions was calculated, and subsequently transformed to z-maps using Fisher r-to-z transformation. The Z-matrix values conformed to a Gaussian distribution, and the data was subsequently examined for outliers before proceeding with statistical tests. A network-based statistics (NBS) approach was then used to identify the whole-brain functional connectivity difference between subjects administered with OT compared to PLC with head motion effects measured as the mean framewise displacement (FD) and age included as a covariate (ANOVA, FWE < 0.05).

### Graph estimation and network characterization

The z-transformed connectivity matrices were utilized to construct brain graphs, which represented the functional connectome for each subject. Subsequently, the functional network properties for each subject were calculated based on the corresponding functional connectivity matrix. The functional connectivity metrics were binarized to generate undirected adjacency matrices, wherein the correlation above the proportional threshold was set to 1, and 0 otherwise. This binarization was performed to include only the strongest functional network connections. Subsequently, a variety of sparsity thresholds were implemented on the correlation matrices, ranging from 10% to 50% in increments of 1%. This was done to facilitate accurate estimation of small-world propensity parameters and to reduce the occurrence of false edges, as suggested by Feng et al. (2012). For each individual participating in this study, we defined sparsity as the proportion of present edges within a network. Initially, the mean connectivity matrix representing the two treatment groups was computed. Subsequently, both global and nodal network metrics were computed for each level of sparsity.

The global network metrics examined in this study included clustering coefficients, characteristic path lengths, and network efficiency parameters, specifically global efficiency and local efficiency. In addition to these traditional metrics, we computed small-world propensity to assess how each node within the network balances local clustering with short path lengths. Small-world propensity quantifies small-world-like properties by comparing each node’s local clustering coefficient and local path length to those of a random network (Telesford et al., 2011). This measure provides insight into the local efficiency and integration of each node, revealing how individual regions contribute to the broader network organization. A node with high small-world propensity demonstrates strong local clustering and short path lengths to other nodes, reflecting its ability to efficiently communicate with distant regions of the network.

Similarly, the nodal network metrics, such as nodal efficiency, nodal degree, and nodal betweenness centrality, were also computed. However, the graph measures of interest to investigate our hypothesis were the small-world propensity measures, clustering coefficients, characteristic path lengths, nodal path, efficiency (local and global), and nodal degree. While these selected measures prevent redundancy since some of these metrics exhibit relationships with one another, either proportionally or inversely, they also provide comprehensive view of brain network organization, each capturing a distinct aspect of brain function. Mathematically, these key metrics are presented as follows:

### Global efficiency ( $E_{glob}$ )

Global efficiency measures the efficiency of information exchange across the entire network. It is the average of the inverse shortest path lengths between all pairs of nodes (Wang et al., 2009).

$$E_{glob}=\frac{1}{N\left(N-1\right)}\sum_{i\neq j\in G}\;\frac{1}{d_{ij}}$$

where by N is the number of nodes in the network and $d_{ij}$ is the shortest path length between nodes i and j .

### Local efficiency ( $E_{loc}$ )

Local efficiency quantifies the efficiency of communication within the local neighborhood of a node (Hayasaka and Laurienti, 2010).

$$E_{loc}=\frac{1}{N}\sum_{i\in G}\;E_{glob}\left(G_{i}\right)$$

where by $E_{glob}\left(G_{i}\right)$ is the global efficiency computed for the subgraph $G_{i}$ containing only the neighbors of node i .

### Clustering coefficient ( C )

The clustering coefficient measures the tendency of a node’s neighbors to be connected to each other. A high clustering coefficient indicates that neighboring nodes form tightly knit clusters or triangles (Sporns et al., 2007)

$$C=\frac{1}{N}\sum_{i\in G}\;\frac{2T_{i}}{k_{i}\left(k_{i}-1\right)}$$

where by $T_{i}$ is the number of triangles involving node i and $k_{i}$ is the degree of node i (number of edges connected to i ).

### Small-world propensity $\left(\gamma_{i}\right)$

The Small-World Propensity $\left(\gamma_{i}\right)$ quantifies the degree to which a given brain region (node i ) exhibits small-world properties, which are characterized by high local clustering and short path lengths relative to a random network (Bassett and Bullmore, 2006). This measure was computed for each node using the following formula:

$$\gamma_{i}=\frac{C_{local,observed\left(i\right)}}{C_{local,random\left(i\right)}}\times \frac{L_{local,observed\left(i\right)}}{L_{local,random\left(i\right)}}$$

where by $C_{local,observed\left(i\right)}$ is the local clustering coefficient of node $\left(i\right)$ in the observed network and $C_{local,random\left(i\right)\;}$ is the local clustering coefficient of node $\left(i\right)$ in a random network with the same number of nodes and edges.

Again, $L_{local,observed\left(i\right)}$ is the local path length of node $\left(i\right)$ in the observed network and $L_{local,random\left(i\right)}$ is the local path length of node $\left(i\right)$ in the random network.

### Nodal degree ( $k_{i}$ )

The degree of a node is the number of direct connections (edges) it has to other nodes (Zalesky et al., 2010).

$$k_{i}=\sum_{j\in G}\;A_{ij}$$

where by $A_{ij}$ is the adjacency matrix element, indicating whether nodes i and j are connected ( $A_{ij}=1$ ) or not ( $A_{ij}=0$ ).

### Characteristic path length ( L )

The characteristic path length is the average shortest path length between all pairs of nodes in the network (Van Wijk et al., 2010).

$$L=\frac{1}{N\left(N-1\right)}\sum_{i\neq j\in G}\;d_{ij}$$

where by $d_{ij}$ is the shortest path length between nodes i and j . For more details, a recent review by Rubinov and Sporns (2010) offers comprehensive definitions and descriptions of these metrics.

**FIGURE 1:**
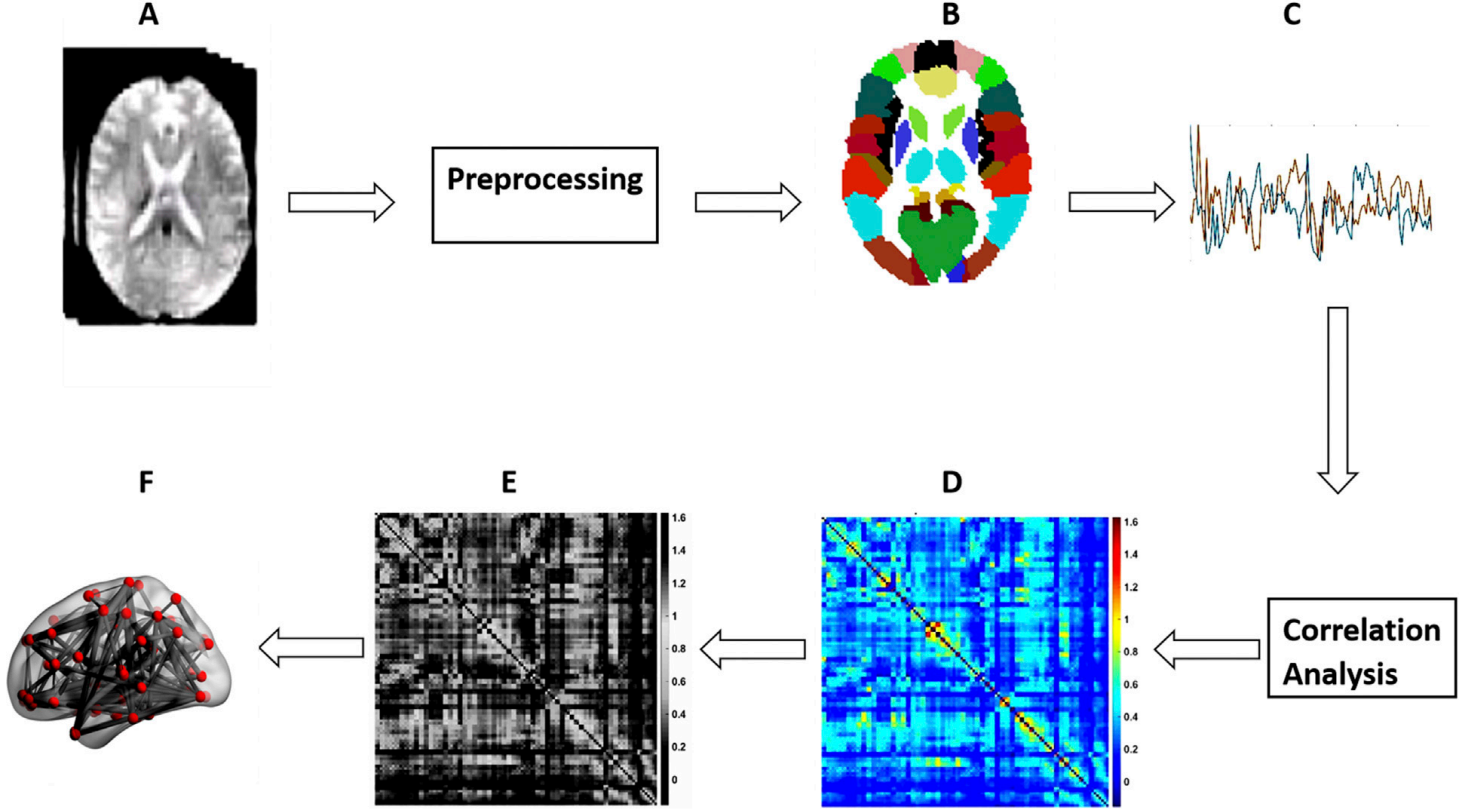
Steps for schematic network analysis. **(A)** rs-fMRI; **(B)** Parcellation; **(C)** Time Series Extraction; **(D)** FC matrix Construction; **(E)** FC Matrix thresholding; **(F)** Brain graph calculation.

The GraphVar toolbox was employed for the various graph measures computations (Kruschwitz et al., 2015; Waller et al., 2018). Additionally, the area under the curve (AUC) was used to summarize each possible value for each of the metrics analyzed on the graph (Pickering and Endre, 2012). This allowed us to generate short estimates for each graph metric that are sensitive to topological variations in brain networks and independent of the selection of a single threshold. Therefore, we used AUC parameters instead of raw values for the entirety of our statistical analyzes with graph measures. This process is illustrated diagrammatically in Figure 1.

### Statistical analysis

We tested for significant differences in FC between the OT and PLC groups at the level of individual brain connections. This was achieved through univariate testing, which assessed the connectivity of each individual link within the brain’s functional network. To control for multiple comparisons, we applied Network-Based Statistics (NBS). This method identified clusters of brain regions with significantly altered connectivity between the two groups, allowing us to detect subnetworks showing FC differences while minimizing false positives.

To further strengthen the reliability of our results, we conducted permutation testing, where the group assignments (OT vs. PLC) were randomly shuffled across 1,000 iterations. This approach compared the observed FC differences to the distribution of differences obtained from the randomizations, ensuring that the observed results were not due to chance and bolstering confidence in their robustness. Then, we examined differences in network topology between the OT and PLC groups by applying sparsity thresholding ranging from 10% to 50%. This approach allowed us to test how the treatment effects on brain connectivity varied across different network densities, ensuring that our results were not influenced by a specific threshold. Finally, to account for multiple comparisons and reduce the risk of Type I errors, we employed FDR corrections, with results considered significant only if the corrected *p*-value was *p* < 0.05.

Additionally, two sample two tailed t-tests were employed to estimate the between group differences in nodal metrics. 1,000 permutations were performed on the AUC of the network metrics through a permutation based non-parametric test. FDR correction for multiple comparisons was then used to identify findings that achieved a significance of *p* < 0.05.

## Results

### Subject demographics and behavioral measures

The analysis of the questionnaire data revealed that there were no significant differences in age, psychological traits or mood indices between the treatment groups. Independent sample t-tests revealed no significant disparity in the mean FD in head motion between the treatment groups. Three participants needed to be excluded due to their failure to attend all of the experimental session resulting in a final sample consisting of 139 healthy male participants (See Table 1).

**TABLE 1:** Demographics and clinical characteristics data.

Variables (mean ± SD)	OT	PLC	t-value	*p*-value
No. of subjects	67 (Male)	72 (male)		
Age	22.09 ± 2.44	21.69 ± 2.57	0.931	0.353
EQ score	37.87 ± 13.37	40.12 ± 13.68	−0.984	0.326
ASQ score	21.54 ± 8.87	20.21 ± 7.42	0.954	0.338
BDI score	6.15 ± 5.55	5.18 ± 5.59	1.025	0.307
STAI score	39.91 ± 9.85	39.37 ± 9.77	0.364	0.716
PANAS (POS)	27.07 ± 7.92	27.20 ± 7.93	−0.107	0.914
PANAS (NEG)	14.43 ± 5.82	15.05 ± 5.87	−0.653	0.514

Abbreviations: Becks Depression Inventory (BDI), State-trait Anxiety Inventory (STAI); EQ, empathy quotient; ASQ, autism spectrum quotient; Positive and Negative Affect Schedule (PANAS); SD, Standard Deviation; POS, positive; NEG, negative.

### Effects of OT on functional connectivity patterns/network edges

**FIGURE 2:**
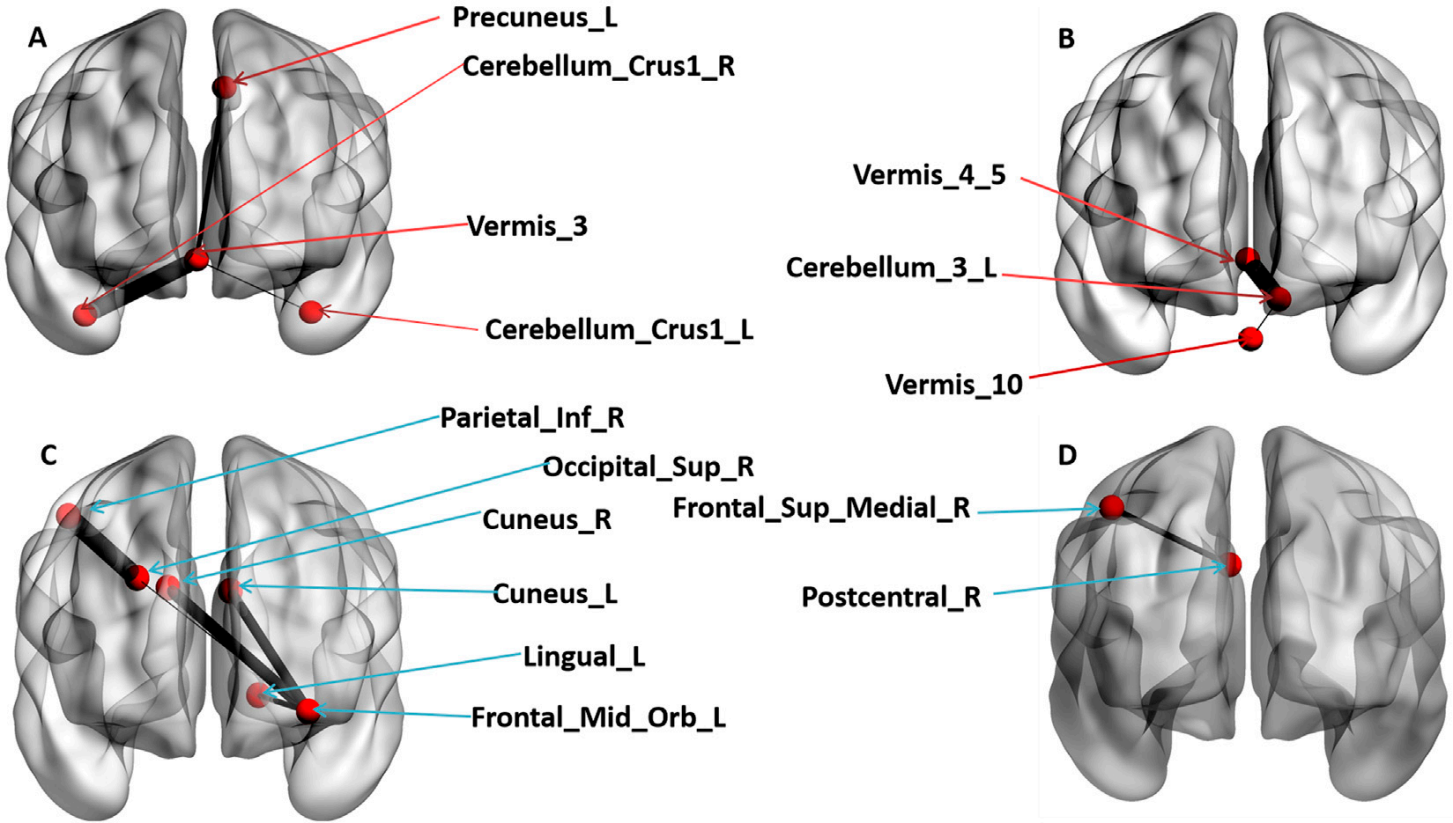
The coronal view of significant Functional connectivity patterns of brain regions that survived multiple FDR corrections (*p* < 0.05). **(A)** denotes increased FC patterns of OT group compared to PLC group. **(B)** denotes increased FC patterns in OT group compared to PLC group. **(C)** denotes decreased FC patterns in OT group compared to PLC group. **(D)** denotes decreased FC patterns in OT group compared to PLC group. The red arrows points to brain regions that revealed increased FC patterns while the blue arrows point to brain regions that revealed a decreased FC pattern.

The influence of OT on FC patterns was examined. Following multiple FDR corrections, significant differences between the OT and PLC groups were observed. The OT group showed increased FC patterns between the cerebellar vermis_3 and the left precuneus (r = 0.098, *p* = 0.001), the cerebellar vermis 3 and left crus1 (r = 0.096, *p* = 0.001) and between the cerebellar vermis 3 and right crus1 (r = 0.105, *p* = 0.001) respectively (see Figure 2A). The OT group also showed increased FC between the left cerebellum 3 and the vermis 4 and 5 (r = 0.120, *p* = 0.001), as well as between the left cerebellum 3 and the vermis 10 (r = 0.118, *p* = 0.001) (see Figure 2B).

Furthermore, the OT group showed reduced FC patterns across interconnected brain regions, including the left middle orbitofrontal cortex and the left cuneus (r = −0.097, *p* = 0.001), left middle orbitofrontal cortex and right cuneus (r = −0.1005, *p* = 0.001), left middle orbitofrontal cortex and the left lingual gyrus (r = −0.090, *p* = 0.001), left middle orbitofrontal cortex and the right superior occipital gyrus (r = −0.084, *p* = 0.001), the right superior occipital gyrus and the right inferior parietal cortex (r = −0.110, *p* = 0.001) and finally between the right posterior central sulcus and the right medial superior frontal cortex (r = −0.095, *p* = 0.001) regions of the brain. Figures 2C, D) presents detailed significant regions. Again, the results were visualized using BrainNet Viewer (Xia et al., 2013).

### Effects of OT on the global and nodal metrics

After multiple FDR correction, we observed the following treatment effects on global and nodal metric measures:

#### Global efficiency

There were no significant treatment effects on the global efficiency measure for the OT relative to PLC group after multiple FDR corrections.

#### Small-world propensity

**FIGURE 3:**
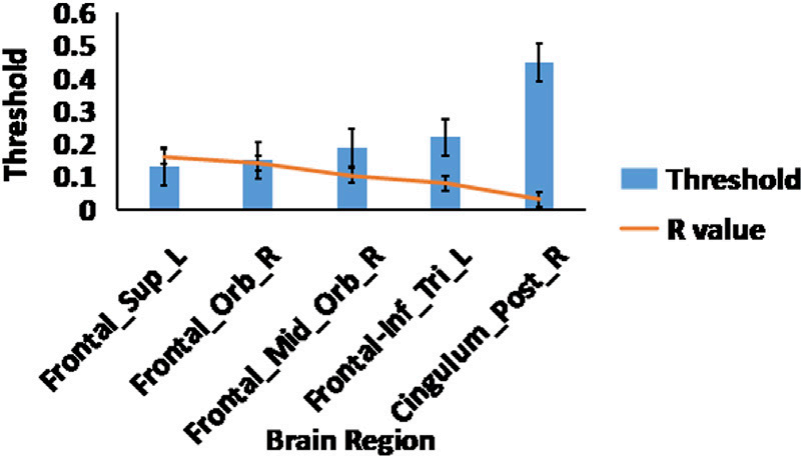
Small world propensity measures. The effects of OT compared to PLC on the small world propensity network metric using 2-sample t-test, After FDR multiple corrections (*p* < 0.05); the displayed brain regions were significant. The blue bars represent sparsity threshold while the orange line represents the R-values.

Analysis of the small world propensity network topology measure revealed significant differences between OT relative to PLC after multiple FDR correction. OT increased the small world propensity topological measures of the right posterior cingulate cortex (r = 0.032, *p* = 0.001), right orbitofrontal cortex (r = 0.142, *p* = 0.001), right superior frontal gyrus (r = 0.161, *p* = 0.001), left inferior frontal gyrus (r = 0.081, *p* = 0.001) and right middle orbitofrontal cortex (r = 0.103, *p* = 0.001) (see Figure 3).

#### Clustering coefficient

Significant differences after FDR multiple correction occurred for clustering coefficients of several nodes of the brain in the OT compared to PLC group. OT increased the clustering coefficient of the cerebellar vermis 3 brain region across different network sparsity thresholds and that of the left Thalamus region (see Table 2).

**TABLE 2:** The effects of OT on the global and nodal topological measures (nodal path, clustering coefficient and local efficiency).

Threshold	Node path	Direction	R-Value	*p*-value (FDR- corr)
0.26	left Caudate	OT < PLC	−0.295	0.001
0.26	Right Caudate	OT < PLC	−0.277	0.001
0.27	left Caudate	OT < PLC	−0.272	0.001
0.27	Right Caudate	OT < PLC	−0.311	0.001
0.30	Right Caudate	OT < PLC	−0.195	0.001
0.32	left Caudate	OT < PLC	−0.178	0.001
0.33	left Caudate	OT < PLC	−0.207	0.001
0.37	left Caudate	OT < PLC	−0.188	0.001
0.38	left Caudate	OT < PLC	−0.225	0.001
0.39	left Caudate	OT < PLC	−0.216	0.001
0.40	left Caudate	OT < PLC	−0.235	0.001
0.41	left Caudate	OT < PLC	−0.201	0.001

Threshold	Local Efficiency	Direction	R-Value	*p*-value (FDR-corr)
0.10	Vermis_3	OT > PLC	0.202	0.001
0.11	Vermis_3	OT > PLC	0.212	0.001
0.12	Vermis_3	OT > PLC	0.214	0.001
0.15	Vermis_3	OT > PLC	0.215	0.001
0.46	Vermis_10	OT > PLC	0.119	0.001

Threshold	Clustering Coefficient	Direction	R-Value	*p*-value (FDR-corr)
0.11	Vermis_3	OT > PLC	0.184	0.001
0.12	Vermis_3	OT > PLC	0.194	0.001
0.23	Left Thalamus	OT > PLC	0.089	0.001

FDR-corr indicates FDR-corrected *p* values, all <0.001. *For healthy males, the topological effect of treatment compared the difference values (OT, minus PLC) between groups.

#### Nodal path

OT relative to PLC significantly decreases nodal path lengths in the right and left caudate regions of the brain across different network sparsity thresholds after multiple FDR correction (see Table 2).

#### Local efficiency

OT relative to PLC significantly increased local efficiency of the cerebella vermis 3 and 10 brain regions after multiple FDR correction (see Table 2).

## Discussion

The objective of the current study was to investigate the pharmacodynamic impact of OT on whole-brain FC network topology in a large cohort of adult male healthy subjects. Utilizing an exploratory and data-driven approach, we aimed to examine differences in pairwise FC patterns at the whole brain level and the effects of OT on small world propensity network topology. By employing graph theory, the study sought to provide insights into the functional brain networks modulated by OT at rest and to clarify the nature of these changes to provide a deeper insight into how OT influences human social behavior and cognition.

### Differences in functional connectivity between OT and PLC

Our key findings revealed that, compared to PLC, intranasal OT modulates inter-regional FC patterns, small-worldness, and regional organization of topographical networks in the resting human brain. We identified that OT’s modulatory effects were associated with widespread brain regions, in frontal, parietal and occipital cortices as well as sub-cortical and cerebellar regions. These regions form networks that enhance information robustness in the human brain during cognitive control and executive functions and are involved in planning, decision-making, working memory, motor control, learning, and emotional regulation. However, our findings showed no significant differences after multiple false discovery rate FDR corrections in the global efficiency topological measure and the mean functional connectivity of OT compared to PLC, regarding global information transfer across brain regions of the human connectome. This is consistent with several previous studies (Poppy, 2014; Santander, 2017; Martins et al., 2021; Zheng et al., 2021).

In terms of altered patterns of resting state functional connectivity intranasal OT administration promoted both increased and decreased connectivity in a number of brain regions in line with some previous findings (Eckstein et al., 2017; Sripada et al., 2013; Zhao et al., 2019b). The most notable increased in functional connectivity particularly involved the cerebellum and included that between the posterior cerebellar vermis and other regions of the cerebellum as well as the precuneus in the default mode network. The vermis, situated within the cerebellum, has recently been shown in animal studies to play an important role in the social brain network (Chao et al., 2021; 2023). The vermis may orchestrate the neural framework essential for Social Recognition Memory (SRM), a process that entails the identification of familiar individuals to establish and sustain social connections (Saito, 2023; Van Overwalle et al., 2020b). Similarly, the cerebellum, while traditionally known for its role in motor control, has been shown in recent research to participate in cognitive functions and emotional responses (Lee, 2023). The increased functional connectivity between vermis 3 and the precuneus may reflect an effect of OT on facilitating judgment of mental states in others (Schmidt et al., 2020; Van Overwalle et al., 2020a). Interestingly, several previous resting state studies have reported effects of OT on cerebellum functional connectivity with one study reporting decreased connectivity with reward areas (putamen) (Zhao et al., 2019b), and another with increased connectivity with the amygdala (Eckstein et al., 2017) which might suggest differential effects on reward and emotional control.

On the other hand, we found evidence for reduced functional connectivity between the medial orbitofrontal cortex and visual processing regions (cuneus, lingual gyrus, superior occipital gyrus) as well as the inferior parietal cortex. This circuitry may represent risk taking circuitry (Rolls et al., 2022) and intranasal OT has been reported to reduce risk taking (Patel et al., 2015).

### The effects of OT on global and nodal metrics

The human brain’s topological characteristics, such as hubs, small-worldness, and network efficiency, are of great functional importance (Bullmore and Sporns, 2009; Bassett and Bullmore, 2009). The small-world network, as proposed by Bullmore and Sporns (2009), is an optimized and highly efficient network model that supports cognitive fitness, functional segregation, and information processing integration. It balances short path lengths and high local clustering to cater to both global and local processing needs (Kaiser and Hilgetag, 2006). While the small world propensity metric quantifies how closely a network resembles this small-world structure, characterized by high clustering and short path lengths, we found in the current study that OT, compared to PLC, enhanced the small-world propensity of the network, particularly in frontal cortical regions including the right orbitofrontal cortex, right middle orbitofrontal cortex, left inferior frontal gyrus and the superior frontal gyrus. This finding is opposite to that reported by Zheng et al. (2021) but may reflect their small sample size. Oxytocin also enhanced small-world propensity network topology in the posterior cingulate cortex, a highly evolved brain region responsible for higher cognitive functions such as thinking, planning, and decision-making. These frontal and parietal regions play a vital role in complex cognitive control and executive functions, including planning, decision-making, working memory, and cognitive control. This aligns with previous studies indicating that abnormalities in these brain regions typically result in functional cognitive decline (Dickerson and Sperling, 2008; Christopher and Strafella, 2013). Dysfunctions in these areas are also often associated with developmental disorders such as attention deficit hyperactivity disorder and autism spectrum disorder, where executive function deficits are prevalent. As a result, the modulatory effects of intranasal OT administration on these critical brain regions further support its potential as a therapeutic agent for neurodevelopmental disorders.

The graph metric known as the “clustering coefficient topology” is utilized in network analysis to quantify the interconnectedness among a node’s neighbors in the context of brain networks. OT increased clustering coefficient topology in the study suggesting that it may enhance the local interconnectedness of neurons in the brain, potentially leading to more efficient information processing within local networks. However, it is important to note that the specific effects can vary depending on the context and the individual’s overall health and mental state. Evidence suggests that brain networks possess a high clustering coefficient, indicating that neighboring regions of the brain are likely to be interconnected (Yin et al., 2017). Specifically, OT increased clustering coefficient in the vermis and thalamus, which are part of complex networks and contribute to various cognitive and motor functions. The thalamus, which is globally connected with distributed cortical regions, serves as an integrative hub of the functional network. Similarly, the vermis, which is associated with bodily posture and locomotion, is part of the spinocerebellum system and receives somatic sensory input from the head and proximal body parts via ascending spinal pathways (Hordacre and McCambridge, 2018; Kaut et al., 2020; Loutit et al., 2021). Recent studies indicate that the cerebellar vermis plays a role in managing a specific aspect of social memory in mice, highlighting its function in the brain’s social network (Chao et al., 2021; 2023). Consequently, an increase in the clustering coefficient suggests that OT may enhance the local interconnectedness of neurons in the brain, potentially leading to more efficient information processing within local networks.

Local efficiency, a key metric in network analysis, measures the efficiency of information transfer in each node’s neighborhood. Evidence suggests that an increase in local efficiency signifies high fault tolerance, as it indicates that the network remains well connected when any node is removed. In the current study, we found that OT increased the local efficiency network topology in vermis 3 and 10 brain regions at different sparsity thresholds, compared to PLC. This aligns with a recent study from Martins et al. (2021), which reported an increase in the local efficiency measure of the brain regions related to the cerebellum, right cuneal cortex and the right posterior superior temporal gyrus. For a healthy brain to exhibit small-world properties, an increase in local efficiency and the clustering coefficient would reveal a more optimal balance between local specialization and global integration, facilitating efficient information processing and robustness against damage.

Furthermore, we observed that OT, compared to PLC, decreased the nodal path length basal ganglia brain reward regions (left caudate and right caudate) at different sparsity thresholds. The nodal path length in a network is a measure of the average shortest path between a node and all other nodes in the network. A decrease in this measure could indicate more efficient information transfer within specific brain regions. The caudate nucleus is involved in various cognitive and emotional functions, but is primarily known for its role in reward, and plays a pivotal role in various higher neurological functions including planning the execution of movement, learning, memory, reward, motivation, emotion, and romantic interaction (Schultz, 2016; Takakusaki et al., 2004). Overall therefore, OT may be promoting more efficient information transfer with the caudate to facilitate its functions, and has been shown to modulate responses to socially rewarding stimuli in this region (Kou et al., 2021; Scheele et al., 2013).

## Limitations

Several limitations should be acknowledged in the context of the current findings. Firstly, only male participants were included in the current study in line with most other resting state studies and therefore it is possible that OT may have different effects in females and this will need to be confirmed in future studies. Secondly, we did not include resting state fMRI scans within subjects before and after OT administration, as suggested by Wu J. T. et al. (2020), which might have produced different results. Lastly, we focused on static network alterations rather than constructing a temporal dynamic network model after OT administration. This limitation prevented us from gaining further insight into the modulatory effects of OT on causal relations. Therefore, we suggest that future research should employ a more dynamic approach to better understand the effects of OT on brain networks.

In conclusion, findings in the current study revealed that intranasal OT administration induces alterations in functional connectivity patterns and enhances small-world topological networks within the social brain of healthy adult males. These results suggest that intranasal OT possesses pharmacodynamic properties capable of modulating connectivity and organization in the social brain to improve its processing efficiency. These findings provide further support for the development of OT administration as potential therapeutic strategy in disorders with social dysfunction. However, further research is necessary to fully elucidate the underlying mechanisms and clinical implications of these brain network effects.

## Data Availability

The raw data supporting the conclusions of this article will be made available by the authors, without undue reservation.
